# Views of professional stakeholders on readiness for a safe road system in Nepal; an exploratory qualitative study

**DOI:** 10.1080/17457300.2021.1983845

**Published:** 2021-09-30

**Authors:** Bidhya Pandey, Anish Khadka, Elisha Joshi, Sunil Kumar Joshi, John Parkin, Paul Pilkington, Julie Mytton

**Affiliations:** a Nepal Injury Research Centre, Kathmandu Medical College Public Limited, Bhaktapur, Nepal; b Centre for Transport and Society, University of the West of England, Bristol, UK; c Centre for Public Health and Wellbeing, University of the West of England, Bristol, UK

**Keywords:** Road traffic injury prevention, safe systems, stakeholders’ perception, thematic analysis, exploratory study, Nepal

## Abstract

Road traffic injuries in Nepal are increasing despite being largely preventable. Little evidence exists regarding the barriers and facilitators to a safer road system. This study aimed to explore the perspectives of professionals whose jobs had the potential to influence road safety in Nepal regarding challenges and potential solutions. Semi-structured interviews with eight informants from diverse roles were analysed thematically. Three themes were identified: Modifying behaviours of road users; Road planning, construction and maintenance; and the Governance of roads and traffic. All participants considered the primary cause of crashes to be the negligent behavior of the road users, suggesting that improved knowledge would influence their decisions. Poor road design, building and maintenance, together with poor vehicle standards, and lack of investment and enforcement of existing road safety legislation, needed to be addressed through greater coordination of the agencies. The study identified a range of areas for future inquiry and action.

## Background

Road traffic injuries are an important public health problem in Nepal with annual increases in numbers of victims (Karkee & Lee, 2016). The Global Burden of Diseases study ranked road injuries as the seventh leading cause of mortality in Nepal in 2017 (Pant et al., 2020). The World Health Organization (WHO) has estimated Nepal’s road traffic fatality rate as one of the highest in South-East Asia at 17 per 100,000 (WHO, 2016). The WHO promotes a ‘safe systems’ approach to road safety. A safe system is one in which the likelihood of events leading to road traffic crashes (RTCs) with fatalities and severe injuries is reduced, and the harm caused by individual road users’ mistakes is minimized, due to the collective actions of multiple sectors of society working together (World Road Association website [PIARC], 2019). Following the announcement of the first Decade of Action of road safety in 2011, the WHO recommended action across five ‘pillars’ of road safety; road safety management, safer roads and mobility, safer vehicles, safer road users and having an effective post-crash response system (WHO, 2011). This approach to road safety is now being further developed into the safe system approach (Welle et al., 2018). Understanding the views of those responsible for the design, construction, maintenance and management of roads and stakeholders involved in traffic management and safety may improve knowledge of the contexts of crashes and provide opportunities for interventions. This is particularly important currently in Nepal, when there is significant expansion and upgrading of road infrastructure across the country (Department of Roads, Ministry of Physical Infrastructure and Transport, 2018). In addition, the number of registered vehicles is rapidly increasing in Nepal (from 1.8 million to 3.5 million over the period 2013/14 to 2018/19) (Ministry of Finance, 2019). Additional road networks and vehicles have the potential to increase the risk of road crashes. There is a lack of existing data and little research has explored the views of stakeholders regarding the road system in Nepal (Pant et al., 2014). Therefore, the aim of this study was to improve our understanding on the perceptions, experiences and beliefs of professionals whose roles enable them to contribute to enhanced road safety.

## Methods

### Study design

This exploratory study was conducted to initiate understanding of the perceptions, experiences and beliefs of people with varied professional backgrounds regarding factors that influence road safety in Nepal. We conducted a qualitative study using semi-structured key informant interviews to gain new insights on common concerns and potential areas for action to improve the road safety systems.

### Study setting

The study was conducted in two districts of Nepal (Kathmandu and Makwanpur), that provide a mixture of urban and rural contexts. The research team had existing professional and personal contacts in both locations to facilitate access to potential participants. In Makwanpur district, this was reached through a well-established NGO with strong links to the local community.

### Participants

We purposively selected participants from a range of professions including both those in public-facing roles (such as the police, driving training centre staff) and those with strategic decision-making authority (such as local and national staff employed to address road traffic management or road construction). Recruitment focused on including participants whose job responsibilities had the potential to impact on the management of road risks or the provision of care to people injured in road crashes. Potential participants were provided with a participant information sheet and met in person to explain the study and answer questions. Informed consent was taken prior to data collection.

### Data collection

Face-to-face interviews were conducted in Nepali by the second and third authors; with one researcher facilitating the interview and the other taking contemporaneous notes. The interviews were conducted at a time and location negotiated with each participant; all interviewees chose to have their interview at their respective workplace. We developed and used a topic guide to focus the interview and encourage participants to contribute their views on the following issues: population groups vulnerable to road traffic injuries; the main causes of road crashes; and the roles and responsibilities of their organisation, and other organisations, on road safety. With permission, interviews were audio-recorded. Data collection continued until a diverse range of views had been collected.

### Data analysis

Interview recordings were transcribed verbatim and translated into English, with participants anonymised by replacing their names with a unique identifying code. The first author checked the interview transcripts against the Nepali audio files to assure the quality of the translation and that meaning had been retained. Data were uploaded into NVivo-12 data analysis software (QSR International) and analysed thematically, using the six step approach described by Braun and Clarke (Braun & Clarke, 2013). Thematic analysis was chosen to enable the identification, inductively and flexibly, of patterns within the interview data, and to generate themes that would indicate areas for potential future action. We followed an iterative process of going forth and back from transcript to code and vice versa, to ensure that the developed codes corroborated the transcripts. Two translated transcripts were double coded by the first and last author to develop a coding framework which was then applied to the remaining transcripts. We used visual maps to collate codes (Braun & Clarke, 2013, p. 232) and to explore the interrelationships between different groups of codes. We subsequently collated codes into broader categories leading to the development of themes that represented the collective views of the participants.

### Ethical consideration

The study received ethical approval from the Institutional Review Committee at the Kathmandu Medical College (Reference number: 310620191) and the Health and Applied Science Faculty Research Ethics Committee of the University of the West of England, Bristol (Reference number: HAS-NAM-16-024).

## Results

Eight participants (6 male and 2 female) were interviewed between October 2019 and May 2020. The profile of the interview participants is presented in Table 1. One participant asked not to have the interview audio-recorded, as he felt it would not be allowed in their organisation. For this participant detailed contemporaneous notes were taken instead. We started to identify repetition within the information arising from the six participants, and added a further two interviews. As this was an exploratory qualitative study we were not seeking data saturation but an indication of issues arising across professional groups. Interviews lasted between 40 and 50 minutes.

**Table 1.** Profile of participants in the key informant interviews.

Participant code	Department/Organisation, Location	Designation/Role
P1	Traffic Police Department, Hetauda	Traffic Police Officer
P2	Road Division Office, Hetauda	Engineer
P3	Transport Management Service Office, Hetauda	Administrator
P4	Driving Training Institute, Hetauda	Driving Instructor
P5	General Police Department, Hetauda	Police Officer
P6	Hetauda Sub Metropolitan City	Health Coordinator
P7	Asian Development Bank Project Directorate Office, Department of Roads, Kathmandu	Project directorate member
P8	Road Development Consultancy, Kathmandu	Highway Consultant

**Figure 1.**
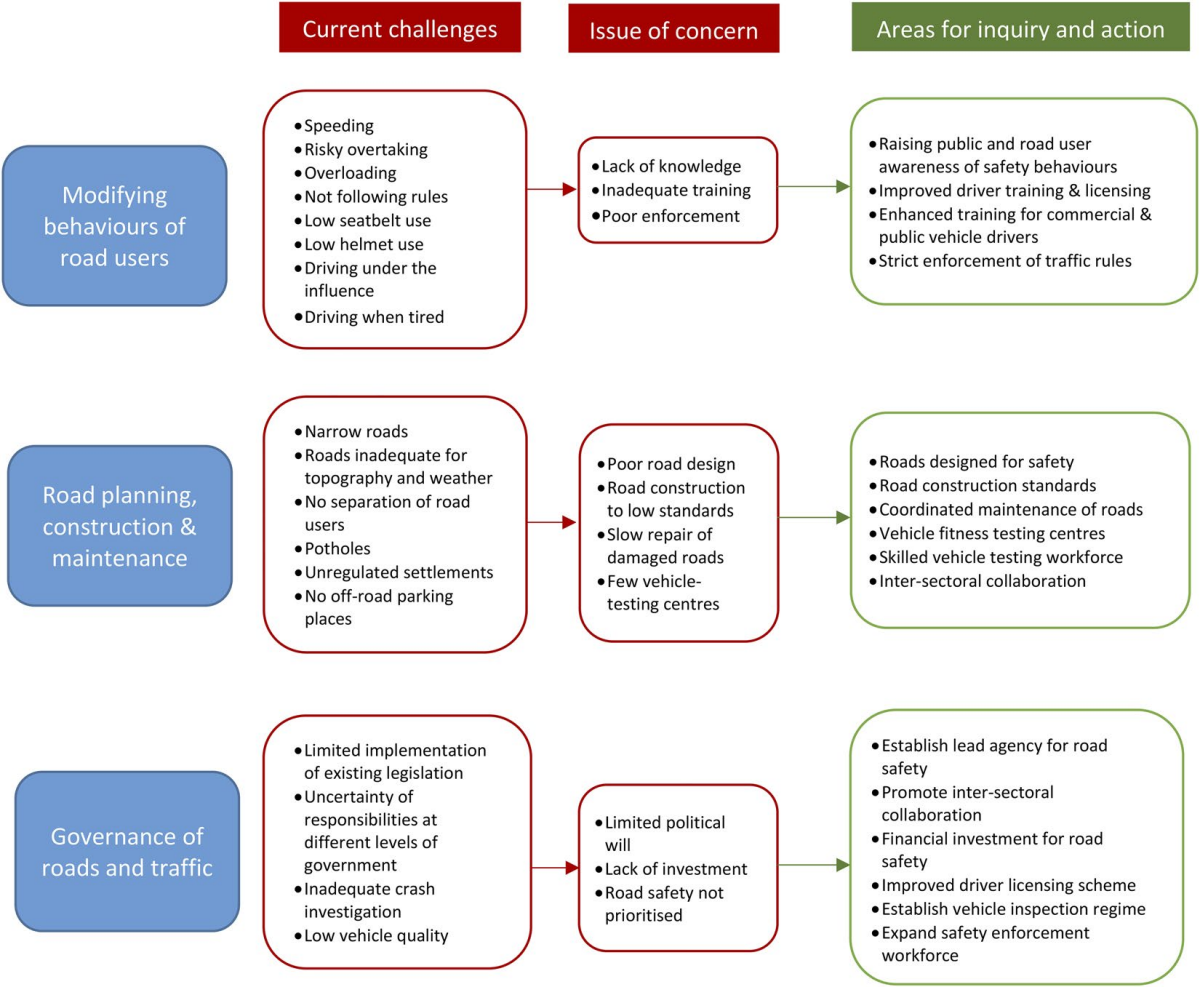
Issues and areas for action identified by participants.

We identified three themes; ‘Modifying behaviours of road users’, ‘Road planning, construction and maintenance’ and ‘Governance of roads and traffic’. Within each theme, participants’ observations of issues that could lead to increased road traffic crashes, and actions that could be taken to improve road safety were identified (Figure 1). Participants tended to use the word ‘accidents’ to describe road traffic crashes and injuries; therefore although this term is no longer routinely used in injury prevention research (Davis & Pless, 2001) the word does appear in the participants’ quotes.

### Modifying behaviours of road users

#### Risky behaviours of road users

All the participants perceived that the behaviour of road users (all types of road user, including pedestrians), was the main cause of RTCs. Participants described speeding, dangerous overtaking, crossing the road in front of oncoming traffic, overloading of commercial vehicles and overcrowding of public vehicles as examples of road user behaviours that increased danger. Participants felt that these behaviors resulted due to a lack of knowledge regarding the safe use of road space, or inadequate training and experience among road users.

All participants described how pedestrians and drivers lack road discipline and do not follow traffic rules, while the passengers tend to compromise on safety while traveling, all of which can lead to RTCs. The traffic police, engineer and driving instructor highlighted that there is a lack of awareness of the traffic rules among the drivers. The traffic police informed that majority of drivers did not comply with the use of seat belts risking their lives during an event of crash. Similarly, the Highway Consultant cited an example on, how pillion motorcycle riders who did not wear helmets were more likely to experience worse injury outcomes. The engineer from the Road Division highlighted that all road users should experience strict enforcement of the laws and regulations.

The culprit for road accidents are the drivers or any road users. A driver is given a lot of different information about vehicles…He is advised not to drink and drive. While riding a motorcycle there are safety issues so they are advised to use helmet and fasten the helmet strap. They are told to obtain enough information about roads. They should also concentrate on road signs present on the roads. Road signs make them aware of the difficulties that lie ahead on the road. If they even give a little bit of attention to traffic signs, road accidents would decrease. I believe it’s the fault of the road user. (P1, Traffic Police)

If pedestrians walk on their side there is a lesser chance for an accident. Crossing the road recklessly even after noticing that a vehicle is approaching, not using zebra crossings are a few examples where an accident occurs because of the pedestrians. (P4, Driving instructor)

Passengers are also faulty; they take ride in a bus even when it is crowded. Vehicle owner and transport committee also make a mistake; hiding the available vehicles, they create artificial shortage of vehicle. Pedestrians and motorcycle drivers also do not follow the rules; they use helmet just to show the traffic police. No one is disciplined. (P5, Police officer)

One of my wealthy friend told me that, his son asked him to buy a bike or else he said he will die. If he bought him a bike, his son would die because of the speed in which he rides the bike and condition of the road…. (P2, Engineer)

Six participants (Traffic police officer, Engineer, Police officer, Health co-ordinator, Project directorate member and Highway consultant) also recognized that the risk of crashes could be reduced by the presence and appropriate use of safety measures such as seat belts, as well as reflectors and helmets on bicycles. The Police officer described motorcyclists do not follow the rules; they use helmet just to show the traffic police.

I think the cyclist should wear reflective clothing… should use reflective lights behind the cycle so that people can see them. This can reduce accident. They should use helmet and gloves… should use protective equipment so that even if there is an accident that minimizes the risk of injury. (P7, Project directorate member)

#### Lack of knowledge

Two participants; the Transport Management Service administrator and the driving instructor, described that professional drivers (e.g. haulage drivers) had relatively low levels of education, often being from rural areas and lacking formal education. Long route bus drivers were also identified as exhibiting risky behaviours such as not taking rest breaks and using alcohol while driving. They were described as being over-confident and lacking patience by the driving instructor and the Project Directorate member.

During festive season, these drivers think of earning more money. The drivers drive without taking rest. They drive without thinking about their health. That is why it causes accidents. (P6, Health coordinator)

I have heard that most of the accidents are due to heavy vehicles like trucks, tippers. Young people of 22 -25 years old drive vehicles in high speed. They drive without any control and the vehicles are loaded heavily. (P3, Transport Management Service Administrator)

Young drivers were viewed by all the participants at high risk of having RTCs. It was known from the participants that youth are keen to learn to drive motorcycles, but less likely to ride responsibly.

Sometimes teenagers drive even when they are drunk. They usually gather in a group of friends, drink alcohol and show stunts with vehicles. Due to these behaviors, number of motorcycle accidents occur. (P1, Traffic police)

Two participants (the Transport Management Service administrator and the driving instructor) claimed that drivers were not trained properly, with the driving instructor describing how drivers stated they were given driving licenses without an assessment of their ability to drive on roads with other traffic or on a variety of types of road (e.g. urban roads, highways, rural roads). Participants from the Department of Transport Management and the driving institute recommended on the need of improving the rigor of the driving license test could help reduce RTCs.

Driving skill is not evaluated in the natural conditions, so even after passing the test, a driver may not be able to drive on the road. (P4, Driving Instructor)

#### Raising awareness of how to keep safe on the roads

Participants identified many ways to promote a sense of social responsibility and safe road use behavior. Seven participants thought raising awareness of traffic rules was paramount, with three (the Transport Management Service administrator, driving instructor and health coordinator) specifically mentioning this was necessary for professional drivers. The Engineer and Project directorate member felt that school children should be made aware of traffic rules in-order to tackle the risky behavior of drivers and pedestrians.

The awareness education should be included on the school curriculum. In many schools of Kathmandu valley have this awareness education in their curriculum which shows and explains the meaning of different traffic signs, rules and regulations. Children will get an opportunity to learn from a small age. (P2, Engineer)

The Engineer reported that the Department of Roads had developed a ‘Road User Guide’ that had been disseminated to improve awareness of road dangers for the general public. Other suggestions included awareness workshops, street drama, and advocacy, using social media.

We should give information on how to reduce road crash from social media, we should encourage people to follow traffic rules. It is because most of the people in Nepal have access to social media; television, internet. Along with aids in media, we can show awareness programs. I think in this way we can reduce road crash. (P7, Project directorate member)

All seven participants who thought awareness programmes were important, emphasised the need for the continuity of such programmes. The Health co-ordinator described one such programme;

We celebrate road safety week. At that time, the local youth clubs conduct awareness programmes on topic like; crossing road from zebra crossing, walking on footpath, using seat belt while driving… They also explain about using helmets while riding. (P6, Health coordinator)

### Road planning, construction and maintenance

The second theme identified concerned the planning, construction and maintenance of roads and how these factors contributed to an increased risk of RTCs.

#### Inter-sectoral collaboration

Three participants (Transport Management Service administrator, Project directorate member and Highway consultant) placed great importance on the need for inter-sector collaboration to plan, construct and maintain the road network. All levels of the federalised government of Nepal (local, provincial and national) needed to take action to make the roads safer. One participant explained how, despite identifying the need for action, there was a lack of prioritisation of road safety and political will.

Positive result can only be obtained when combined efforts of all stakeholders meet at a common point. (P8, Highway consultant)

It should be done by government. We identified problems and show the solutions…we had provided report to the province government but they didn’t implement. This is a main barrier. It is not implemented from political level. (P3, Transport Management Service, senior administrator)

#### Poor road design and management

Narrow roads were identified by six participants as the cause of crashes and this was often because of either vehicles being parked on the roadway or footways not having been constructed. Four participants expressed concern that roads were not built to international standards, the project directorate member said road markings were not regularly repainted and the health coordinator and Highway consultant expressed concerns that barriers were not maintained. Four participants (Traffic police, Transport Management Service Administrator, General police and Health Co-ordinator) reported that the lack of adequate vehicle repair centres along highways where vehicles could be repaired and felt that this could contribute to road crashes. The senior administrator of Department of Transport management service said that the Department of Transport Management is planning to establish seven vehicle fitness centre one in each province along the highways. The participant further elaborated the proposal has been approved in three places Hetauda, Kathmandu and Butwal.

The vehicle fitness centre will have a machine and the vehicle will be placed inside the machine and we can see everything from the screen. It helps to detect problems of engine….But I prefer if there were skilled or trained personnel to look after these things rather than the use of machine. (P3, Transport Management Service, senior administrator)

Five participants (Engineer, Driving instructor, Police officer, Health co-ordinator and Project directorate member) said that damaged roads were not properly maintained timely, increasing the risk of RTCs.

The roads are full of potholes, the elevation of the roads is also not uniform. I was driving in Birgunj [name of a place] a few days earlier and I could not see a pothole on the road and fell into it. The road looks good from distance and when one approach near, it is often too late. (P4, Driving instructor)

Five participants described how road side settlements could contribute to RTCs. The Highway Consultant explained how roadside settlements developed after the roads have been constructed and were unregulated, placing both residents and road users at risk.

In some places, people have followed the traffic rules while in some places, the settlement nearby road; it is unsafe. I have seen that the shops are run on the road that is disturbing both for the drivers and pedestrians….The street vendors want to keep their shop where they desire. In some places, I have seen that the shops are kept in the place which is disturbing both drivers and pedestrians. That is why, everyone is self-oriented ……. (P7, Project directorate member)

The human settlement and the economic activities existing along the east-west highway has developed only after the construction of the highway. The human settlements are dense in major towns and economic areas…. The signs indicating the approach of human settlements, whether it be a town unit or a rural settlement, has to be installed. Currently, we lack a signaling mechanism that can foretell us about the approaching settlements. (P8, Highway Consultant)

The Highway Consultant acknowledged the absence of signs that tell drivers to slow down or that they are entering settlements. The absence of such signs may mean that traffic travels too fast through populated areas.

In some places, near the settlement; it is unsafe…. In the places where there are settlements, there are neither traffic signs nor speed limits and zebra crossings. (P7, Project directorate member)

A common concern of participants was that Nepal has varied geographical terrain and that road construction and maintenance is different in hilly and mountainous areas compared to roads built on the plains. Additional road safety infrastructure is required on hill and mountain roads. In addition, adverse weather such as heavy rain can increase the challenges of road maintenance and ensuring safe mobility, since the rains are commonly associated with landslides.

In most of the places, there is the problem of landslides… I have heard people dying due to fall of stone from the hill. We should have protective barriers in such places. (P6, Health coordinator)

Five participants (Engineer, Transport Management Service administrator, Health co-ordinator, Project directorate member) highlighted separation of lanes for different road users (cyclists, slow speed vehicles, heavy vehicles) and footpaths for pedestrians.

I think in our highway, there isn’t dedicated division of lane. In case of Terai region, there are more cyclists. And as the road isn’t divided into lanes; there is always conflict among the two wheelers and four wheelers. That is why, as far as I am informed from the data, there is more number of accidents among the cyclist during evening or at night. (P7, Project directorate member)

One participant observed that there was poor coordination between agencies and that the roads were repeatedly dug up by different departments. This resulted in potholes which, when not repaired, increased the risk of RTCs.

After constructing road, we again have to dig it for the drain. There is lack of multi-sectoral coordination. The electricity, drain and water pipes should be constructed at the same time. That is why, if there is proper coordination then we can improve. (P6, Health coordinator)

### Governance of roads and traffic

#### Lack of political will

The Transport Management Service administrator and the health coordinator both recognised that frequent changes of government in the recent past had resulted in some previously agreed policies for not being implemented. It was reported by the administrator that the introduction of a federalised system of government in Nepal resulted in uncertainty regarding the responsibilities of the different levels of government with regard to road safety. Five of the participants highlighted a lack of adequate legislation, for example, legislation to determine how blame should be assessed in crash investigations, vehicle safety standards, or lack of enforcement of existing laws and policies that could keep road users safe.

Our country is ruled by people not by the law. Nothing gets changed unless the country gets ruled by the law not by a person…. Unless strong policies, rules and regulations are made, this problem is going to last forever, doesn’t matter how good the road quality is…. (P2, Engineer)

#### Lack of financial investment and resources for law enforcement

One clear complaint was the inadequacy of financial commitment to road safety by the government. This had implications across multiple sectors ranging from incorporating safety features into the design, construction and maintenance of roads, to the ability to enforce traffic legislation using technology such as radar speed guns and breathalysers. Lack of investment has resulted in limited manpower across the relevant sectors including the traffic police, driving instructors and staff to manage a comprehensive vehicle licensing scheme and conduct vehicle inspections.

We don’t have manpower to identify if the vehicle is in proper condition.…. We check condition of tyre, brake and detect whether vehicle emits pollution but we cannot claim that the vehicle isn’t in good condition… (P3, Transport Management Service administrator)

We also check the speed of vehicles sometime… but we don’t have sufficient machines. The machines are really expensive. We don’t have budget… that machine is called radar gun. We have machine called breathalyser which assess… even that machine costs more than one lakh (0ne hundred thousand Nepali rupees)…. One that we have is not functioning… we don’t have technicians who can repair such equipment and we have no other option than storing them. (P1, Traffic police)

Participants suggested that the consequences of violating traffic safety rules should be significant and strictly enforced because this would be a deterrent to other road users. However, one participant (traffic police) highlighted that there is a lack of human resources to enforce traffic laws.

There should be strict punishment. If ‘I do not follow the rule I will be punished’, everyone should be conscious… the laws of our country does not encourage people to follow the rules. (P7, Project directorate member)

We get directions from above (seniors) to inform general public, decrease accidents and to find out the reasons if there are any to decrease accidents. We are limited in manpower and also in resources….The manpower we have right now is very limited…. Actually we have to be present in the related places to decrease the accidents but we have not been able to do that. (P1, Traffic police)

## Discussion

This study has explored the risks and challenges, to reducing road traffic crashes and injuries in Nepal, as perceived by key professionals, and identified their suggestions for opportunities for change. A common perception was that other people, primarily road users or those who build or maintain roads, were those who needed to take action. This is similar to findings of qualitative interviews conducted in Iran by (Khorasani-Zavareh et al., 2009) which concluded that the non-compliant road user as the important factor to RTCs, followed by lack of enforcement of traffic rules, lack of appropriate road infrastructure, and lack of safety features designed in vehicles. Azami-Aghdash et al. (2019), a similar study in Iran reported that their respondents thought that the absence of a responsible agency and lack of inter-sectoral co-ordination was the main barrier for the prevention of road traffic injuries.

A scoping review and a thematic analysis of common road safety approaches adopted by different countries in the world reported three main approaches to road safety; ‘traditional’ approach which describes Road Traffic Injuries (RTIs) as unavoidable and unpreventable, with road users’ responsible for road crashes; secondly, a systemic approach, adopted in many high income countries which considers RTIs as the result of gaps in a road safety system; and thirdly, the vision zero approach, which acknowledges that humans make errors and that the road environment should protect users from these errors leading to injuries (Safarpour et al., 2020). In our study we found that participants largely took the traditional approach; that the dangerous behaviour of road users was considered the most important contributing factor to RTCs in Nepal. All participants thought that speeding was one of the main factors contributing to RTCs, and this is consistent with other findings in similar settings (Khorasani-Zavareh et al., 2009; Tetali et al., 2013).

The introduction of speed limit legislation, especially with physical means of reducing speed such as speed humps, has been found to be effective (Hyder et al., 2012). Participants suggested that dangerous road user behaviour could be tackled by frequent and regular road safety awareness campaigns. This finding is consistent with (Khorasani-Zavareh et al., 2009), however, education alone is unlikely to resolve the issues, and poor road user behaviour can be influenced through careful road planning and design. The stakeholders in our study thought that road planning that incorporated topographically-relevant designs, safety infrastructure and adequate financial investment had the potential to reduce crashes if coordinated at all levels of the federal structure. Participants also suggested that weather was a factor in RTCs, there was also acknowledgement that good road infrastructure could mitigate the risks associated with poor weather which was similar to the findings from a qualitative study done in Vietnam, where participants suggested that inadequate road infrastructure was the main cause of RTCs (La et al., 2017). Participants in our study also thought that different types of traffic should be separated, as well as traffic should be separated from pedestrians e.g. with footways and crossings.

The World Health Organisation advocates for a safe systems approach to road safety that includes formulating policies and strictly enforcing laws related to the use of safety equipment (such as helmets, seat belts and child restraints), drink driving and speed limits (WHO, 2018). In our study, participants frequently mentioned that legislation and enforcement were not strict. Participants reported that driving without a license and lack of adequate training on driving on different types of roads and in traffic prior to getting license, were contributing to RTCs. The WHO emphasizes the value of a standardised and strictly applied driver licensing system (WHO, 2007). This would avoid driving licenses easily being bought, as they are currently in many Low and Middle Income Countries (LMIC), including Nepal (International Federation of Red Cross and Red Crescent Societies, 2007). Participants recognised that there is a lack of human resource and equipment to test vehicle compliance with safety standards. Vehicles in Nepal do not meet an appropriate standard for safety; most vehicles, especially taxis, buses and trucks in LMICs, do not have crash protective designs and features such as air bags or an advanced braking system. Such features are costly for vehicle owners (The World Bank, 2019). Adequate legislation, strict enforcement and availability of the equipment needed to test vehicles were suggested as solutions for improving road safety.

Post-crash response is a supporting component which helps minimize the impact of injury, and can improve injury survivability, or reduce the long term impacts of injury (International Federation of Red Cross and Red Crescent Societies, 2007). Post-crash response is one of the five pillars of road safety, yet none of our participants commented on this issue. With the exception of the respondents to (Khorasani-Zavareh et al., 2009), who mentioned the need for separate lanes for ambulances in busy roads, respondents to similar qualitative studies were also noted to have not raised this issue (Azami-Aghdash et al., 2019; La et al., 2017; Tetali et al., 2013).

In our study, participants described actions that suggested a shift in the road safety paradigm from the traditional to systemic or safe system approach. However, there are still gaps that could be addressed though political will and prioritization. Nepal is a co-sponsor of the UN declarations on road safety so theoretically it has signed up to a safe systems approach, but the reality as suggested by the participants in our study is that action is not joined up. Legislation is incomplete and does not support collective ownership and action (Pant et al., 2021). Despite awareness of road safety issues in Nepal, little governmental action was taken until 2013 (Sustainable Transport and Traffic Solutions, 2015). In 2013, a ministerial level committee was established to develop a Road Safety Action Plan and to determine a lead agency for road safety (The World Bank, 2020a). However, despite an agreement to establish a lead agency, no effective agency has been formed. In 2020, Nepal received a grant from the World Bank to strengthen institutional responses for a safer road system (The World Bank, 2020a). Subsequently, the Government of Nepal drafted a Road Safety Bill; mandating the establishment and responsibilities of the lead agency in Nepal (The World Bank, 2020b). Currently, the bill is at the Ministry of Law, Justice and Parliamentary Affairs for review and will be forwarded in parliament for approval.

The safe systems approach promoted by the WHO, adopted by many high income countries, is now starting to be implemented in cities in LMICs, such as Bogota in Colombia and in Curitiba, Brazil (World Road Association website [PIARC], 2019). Systems thinking helps to shift the focus away from blaming road users to making the transportation system itself the area of attention (World Road Association website [PIARC], 2019). The ‘safe system’ approach to road safety focuses on the inter-relationships of different factors and sectors, such that human behaviour and mistakes should not lead to deaths or severe injuries. Despite international acceptance of the safe systems approach, none of the participants in this study spoke about a safer ‘system’, though they did frequently mention the need for coordinated multi-sectoral collaboration.

### Strengths and limitations

To our knowledge, this is the first qualitative study in Nepal to explore the understanding of professionals working to improve road safety. Participants were from diverse backgrounds in terms of education and work experience, yet they raised similar issues and areas for improvement in relation to road safety. The findings of this study are derived from participants working in Kathmandu and Makwanpur districts. Whilst these areas include both urban, rural and trunk roads and cover topographies typical of many districts of Nepal (plains, low hills and high hills), they may not be generalisable across the whole country, particularly for more rural areas. As this was an exploratory study, we recruited one representative from a range of different professions. Inclusion of more participants from the same professions may have yielded different views. This study has been able to identify a range of issues needing further evaluation and assessment.

## Conclusion

This study explored the perceptions, experiences and beliefs of professionals whose job roles had the potential to improve road safety. The behaviour of drivers and pedestrians was the factor consistently identified by participants as contributing to road traffic crashes. Poor road design, construction and maintenance, limited road safety infrastructure, inadequate safety features of vehicles, poor vehicle maintenance, lack of skilled human resource for driver training and vehicle testing, and poor enforcement of legislation were also identified as challenges to enhancing road safety. Our participants did mention actions that indicate a move towards a safe system, but none of them used that language. This suggests that professional groups may be open to working in a safe systems approach if this was promoted by federal government. Our study suggests that research to understand the awareness between agencies of their complementary roles in road safety, and research to better understand the barriers to a coordinated approach to road safety in Nepal should be undertaken. Such evidence, could inform future strategies to reduce traffic related morbidity and mortality. In order to create the right overall environment for improvements, strong political will is needed to enable action to be taken.

## Data Availability

The data that support the findings of this study are available from the corresponding author, [BP], upon reasonable request.

## References

[CIT0001] Azami-Aghdash, S., Abolghasem Gorji, H., Derakhshani, N., & Sadeghi-Bazargani, H. (2019). Barriers to and facilitators of road traffic injuries prevention in Iran: A qualitative study. *Bulletin of Emergency And Trauma*, *7*(4), 390–398. 10.29252/beat-070408 31858002 PMC6911722

[CIT0002] Braun, V., & Clarke, V. (2013). *Successful qualitative research: A practical guide for beginners*. Sage.

[CIT0003] Davis, R. M., & Pless, B. (2001). BMJ bans “accidents”. Accidents are not unpredictable. *BMJ (Clinical Research ed.)*, *322*(7298), 1320–1321. 10.1136/bmj.322.7298.1320 PMC1120417 11387166

[CIT0004] Department of Roads, Ministry of Physical Infrastructure and Transport. (2018, June). *NEP: SASEC Highway Improvement Project. Initial Environmental Examination. Government of Nepal for the Asian Development Bank*. https://www.adb.org/sites/default/files/project-documents/52097/52097-001-iee-en_0.pdf

[CIT0005] Hyder, A. A., Allen, K. A., Pietro, G. D., Adriazola, C. A., Sobel, R., Larson, K., & Peden, M. (2012). Addressing the implementation gap in global road safety: Exploring features of an effective response and introducing a 10-country program. *American Journal of Public Health*, *102*(6), 1061–1067. https://www.paho.org/mex/index.php?option=com_docman&view=download&alias=789-rs10-introducing-a-10-country-program&category_slug=articulos-de-interes&Itemid=493 10.2105/AJPH.2011.300563 22515864 PMC3483956

[CIT0006] International Federation of Red Cross and Red Crescent Societies. (2007). *Practical guide on Road Safety. A toolkit for National Red Cross and Red Crescent Societies*. International Federation of Red Cross and Red Crescent Societies and Global Road Safety Partnership. Retrieved August 20, 2020, from https://www.ifrc.org/Global/Publications/road-safety/road-safety-en.pdf

[CIT0007] Karkee, R., & Lee, A. H. (2016). Epidemiology of road traffic injuries in Nepal, 2001-2013: Systematic review and secondary data analysis. *BMJ Open*, *6*(4), e010757. DOI: 10.1136/bmjopen-2015-010757 PMC4838689 27084283

[CIT0008] Khorasani-Zavareh, D., Mohammadi, R., Khankeh, H. R., Laflamme, L., Bikmoradi, A., & Haglund, B. J. A. (2009). The requirements and challenges in preventing of road traffic injury in Iran. A qualitative study. *BMC Public Health*, *9*(1), 486. 10.1186/1471-2458-9-486 20030826 PMC2811114

[CIT0009] La, Q. N., Duong, D. V., Lee, A. H., & Meuleners, L. B. (2017). Factors underlying bus-related crashes in Hanoi, Vietnam. *Transportation Research Part F: Traffic Psychology and Behaviour*, *46*(Part B), 426–437. 10.1016/j.trf.2016.06.023

[CIT0010] Ministry of Finance. (2019). *Economic survey 2018/19*. Government of Nepal. https://mof.gov.np/uploads/document/file/compiled%20economic%20Survey%20english%20725_20191111101758.pdf

[CIT0011] Pant, P. R., Banstola, A., Bhatta, S., Mytton, J. A., Acharya, D., Bhattarai, S., Bisignano, C., Castle, C. H. D., Dhungana, G. P., Dingels, Z. V., Fox, J. T., Hamal, P. K., Liu, Z., Mahotra, N. B., Paudel, D., Pokhrel, K. N., Ranabhat, C. L., Roberts, N. L. S., Sylte, D. O., & James, S. L. (2020). Burden of injuries in Nepal, 1990-2017: Findings from the Global Burden of Disease Study 2017. *Injury Prevention*, *26*(Supp 1), i57–i66. 10.1136/injuryprev-2019-043309 31915272 PMC7571348

[CIT0900] Pant, P. R., Mytton, J., Dharel, M. R., Dangi, A., Rai, W. B., Joshi, S. K. (2021). The prevention of – and first response to – injuries in Nepal: a review of policies and legislation. *Health Research and Policy Systems, 19* (65). 10.1186/s12961-021-00686-1 PMC8045995 33853626

[CIT0012] Pant, P. R., Towner, E., Pilkington, P., Ellis, M., & Manandhar, D. (2014). Community perceptions of unintentional child injuries in Makwanpur district of Nepal: A qualitative study. *BMC Public Health*, *14*, 476. 10.1186/1471-2458-14-476 24886124 PMC4031493

[CIT0013] Safarpour, H., Khorasani-Zavareh, D., & Mohammadi, R. (2020). The common road safety approaches: A scoping review and thematic analysis. *Chinese Journal of Traumatology*, *23* (2), 113–121. 10.1016/j.cjtee.2020.02.005 32178997 PMC7156955

[CIT0014] Sustainable Transport and Traffic Solutions. (2015). Development of road safety management system in Nepal. A comprehensive approach for road safety management. (NRSMS). https://www.nrna.org/Portals/0/05%20NRSMS%20BIG%20PICTURE%20Proposal.pdf

[CIT0015] Tetali, S., Lakshmi, J. K., Gupta, S., Gururaj, G., Wadhwaniya, S., & Hyder, A. A. (2013). Qualitative study to explore stakeholder perceptions related to road safety in Hyderabad, India. *Injury*, *44*(4), S17–S23. 10.1016/S0020-1383(13)70208-0[PMC][24377773 24377773

[CIT0016] The World Bank. (2019). *Guide for road safety opportunities and challenges: Low- and middle-income countries country profiles*. Retrieved August 10, 2020, from https://www.worldbank.org/en/programs/global-road-safety-facility

[CIT0017] The World Bank. (2020a). *Government of Nepal and World Bank Sign $450 Million Road Support Project in Nepal to boost post-COVID-19 Recovery*. https://www.worldbank.org/en/news/press-release/2020/07/17/government-of-nepal-and-world-bank-sign-450-million-road-support-project-in-nepal-to-boost-post-covid-19-recovery?fbclid=IwAR2_mzBXbPsGMYAu5oCTg_VLkAbgU_hjQYNoJBvaI2BFMDSykoGmaGqC1XY#:-:text=KATHMANDU%2C%20July%2017%2C%202020%20%E2%80%93,a%20%24450%20million%20

[CIT0018] The World Bank. (2020b). *Delivering road safety in Nepal. Leadership priorities and initiatives to 2030*. Retrieved September 25, 2020, from http://documents1.worldbank.org/curated/en/215411581916828069/pdf/Delivering-Road-Safety-in-Nepal-Leadership-Priorities-and-Initiatives-to-2030.pdf?fbclid=IwAR0g4C62aPi88bKg5_vT0t9MD0cp0vPGgXY8Hhm_AcsNTX9OjMHblDu6lLQ

[CIT0019] Welle, B., Sharpin, A. B., Adriazola-Steil, C., Job, S., Shotten, M., Bose, D., Bhatt, A., Alveano, S., Obelheiro, M., & Imamoglu, T. (2018). *Sustainable and safe: A vision and guidance for zero road deaths*. World Resources Institute. Retrieved August 12, 2021 from https://files.wri.org/d8/s3fs-public/sustainable-safe.pdf

[CIT0020] WHO. (2007). *Youth and road safety*. Retrieved July 20, 2020, from https://www.who.int/management/programme/ncd/Youth%20and%20Road%20Safety.pdf

[CIT0021] WHO. (2011). *Global plan for the decade of action for road safety 2011–2020*. Geneva: World Health Organisation. Retrieved August 12, 2021, from https://www.who.int/roadsafety/decade_of_action/plan/en/

[CIT0022] WHO. (2018). *Global status report on road safety 2018*. Retrieved July 17, 2020, from https://www.who.int/violence_injury_prevention/road_safety_status/2018/en/#:-:text=The%20Global%20status%20report%20on,people%20aged%205%2D29%20years.

[CIT0023] World Health Organization [WHO]. (2016). *Road safety in the South-East Asia Region. Regional office for South-East Asia.* https://www.who.int/violence_injury_prevention/road_safety_status/2015/Road_Safety_SEAR_3_for_web.pdf

[CIT0024] World Road Association website [PIARC]. (2019). *Road safety ­manual. A guide for practitioners on implementing safe system infrastructure*. Retrieved August 2, 2020, from https://roadsafety.piarc.org/en/road-safety-management-safe-system-approach/introduction

